# Restoration of Reproductive Function in Uterine Factor Infertility: Clinical Evaluation of Uterus Transplantation

**DOI:** 10.3390/jcm15145437

**Published:** 2026-07-11

**Authors:** Živilė Sabonytė-Balšaitienė, Karolina Kolosovaitė

**Affiliations:** 1 Clinic of Obstetrics and Gynecology, Institute of Clinical Medicine, Faculty of Medicine, Vilnius University, LT-03101 Vilnius, Lithuania; 2 Faculty of Medicine, Vilnius University, LT-03101 Vilnius, Lithuania

**Keywords:** uterus transplantation, uterine factor infertility, living donor, deceased donor, immunosuppression, clinical outcomes

## Abstract

**Background/Objectives:** According to the World Health Organization, approximately one in six couples experience infertility during their lifetime, accounting for about 15–17% of the reproductive-aged population worldwide. Absolute uterine factor infertility is a condition in which a woman is unable to conceive due to the absence of the uterus or irreversible impairment of its function. Uterus transplantation has emerged as a novel treatment option enabling affected women to achieve biological motherhood. Aim of the study was to review current evidence on uterus transplantation as a treatment for absolute uterine factor infertility, focusing on clinical outcomes, surgical aspects, patient selection, and associated risks. **Methods:** A structured narrative review informed by a literature search was conducted using PubMed, Web of Science, and Scopus databases. Publications addressing clinical outcomes, surgical techniques, donor and recipient selection, immunosuppressive therapy, and ethical aspects of uterus transplantation were analyzed. Publications published between 2016 and 2025 addressing uterus transplantation were reviewed. The selected studies comprised clinical trials and case series, systematic reviews, clinical and methodological analyses, and ethical publications. **Results:** The most common indication for uterus transplantation is MRKH syndrome, although acquired causes such as hysterectomy for oncologic or benign gynecological conditions are also relevant. Patient selection is based on strict medical and psychosocial criteria. Graft survival rates reach approximately 74%, with vascular complications, particularly thrombosis, being the leading cause of graft loss. By 2025, more than 130 uterus transplantations and over 70 live births had been reported worldwide. Clinical pregnancy rates approach 70% among recipients with a functioning graft, while at least one live birth has been achieved in approximately 43% of recipients. Pregnancies following uterus transplantation are considered high-risk and are associated with increased rates of hypertensive disorders, gestational diabetes, preterm delivery, and neonatal complications. Immunosuppressive therapy remains essential but is associated with nephrotoxicity and infectious risks. **Conclusions:** Current evidence indicates that uterus transplantation can restore reproductive function in carefully selected women with absolute uterine factor infertility. Despite encouraging reproductive outcomes, the procedure remains associated with substantial surgical complexity, vascular complications, and immunosuppression-related risks, limiting its broader implementation in clinical practice.

## 1. Introduction

Infertility is a relevant and significant public health issue that has long-term effects on the physical and psychological well-being of individuals of reproductive age. According to the World Health Organization, approximately one in six couples experience infertility during their lifetime, accounting for about 15–17% of the global reproductive-age population [1].

Most cases of infertility can be successfully treated with medical therapy or assisted reproductive technologies. However, for some women, these methods may be ineffective. Common causes include the absence of the uterus or its functional insufficiency. Such fertility disorders encompass conditions in which pregnancy is impossible due to congenital or acquired uterine dysfunction, despite preserved ovarian function and adequate ovarian reserve. This condition is commonly referred to as absolute uterine factor infertility (AUFI) when pregnancy is impossible due to the absence of the uterus or irreversible loss of uterine function. It is diagnosed in 2–17% of women worldwide, depending on the diagnostic classification and the studied population [2]. The causes of uterine infertility in women may be either congenital or acquired. Congenital causes are mostly of genetic origin. One example is Mayer–Rokitansky–Küster–Hauser (MRKH) syndrome, also known as Müllerian agenesis. Congenital causes include MRKH syndrome, uterine agenesis, uterine hypoplasia, and other Müllerian developmental anomalies. Acquired causes of infertility include hysterectomy due to oncological or other surgical reasons, severe intrauterine damage, endometriosis, Asherman’s syndrome, or complications following radiation therapy [2,3].

Before the advent of uterus transplantation, parenthood for women with AUFI was primarily possible through adoption or assisted reproduction with surrogacy. In many countries, surrogacy is prohibited due to associated ethical concerns. Uterus transplantation has emerged as a novel method to provide these patients with the opportunity to conceive and give birth to their biological child. The first successful birth following uterus transplantation occurred in 2014 in Sweden [4]. Following this historic event in medicine, uterus transplantation procedures have expanded to other specialized centers. According to the most recent decade review, by the end of 2024, approximately 130 uterus transplants had been performed worldwide, involving both living and deceased donors, resulting in more than 70 live births [5].

Despite growing clinical interest and increasing experience, uterus transplantation remains a complex, multi-stage, and high-risk intervention. The procedure requires a high level of surgical expertise due to the complex vascular anatomy of the uterus, prolonged operative time, and significant risk of intraoperative complications [6]. The treatment process extends beyond organ transplantation and includes postoperative care, monitoring of graft function, pregnancy planning and management, and immunosuppressive therapy; therefore, uterus transplantation cannot be considered a standard treatment for infertility [6]. Additional challenges arise from the involvement of a living donor. The donor undergoes a major surgical procedure without direct medical benefit. These considerations place particular emphasis on donor safety and informed consent and contribute to the concentration of uterus transplantation programs within highly specialized centers [6,7].

Uterus transplantation is not a life-saving intervention; therefore, careful patient selection and a thorough assessment of the risk–benefit ratio are of paramount importance. The decision to perform this procedure must be based not only on objective medical criteria but also on comprehensive counseling of recipients and donors regarding potential physical, psychological, and social consequences [7]. In modern uterus transplantation programs, not only clinical outcomes but also the long-term well-being of both recipients and donors are important, aiming to ensure the responsible and ethically justified application of this procedure [7].

Considering its clinical potential, complex surgical and therapeutic nature, associated risks, and ethical aspects, uterus transplantation requires thorough scientific evaluation. Therefore, this review aims to summarize current evidence regarding uterus transplantation, including patient selection, surgical techniques, clinical outcomes, associated risks, and ethical considerations, to better define its role in contemporary reproductive medicine.

**Figure 1:**
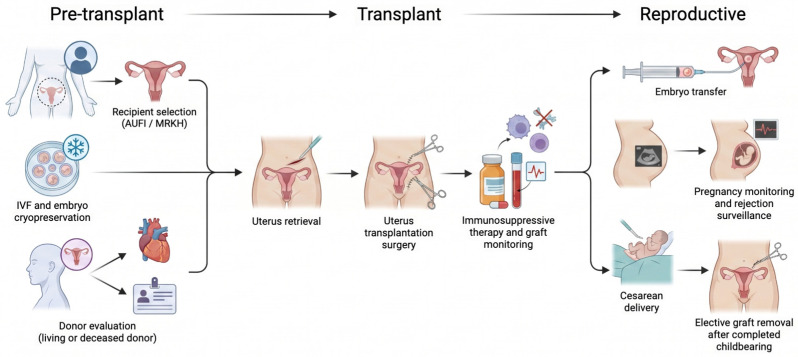
Clinical pathway of uterus transplantation.

The clinical pathway of uterus transplantation is summarized in Figure 1, illustrating the major stages from recipient selection and donor evaluation to pregnancy, delivery, and elective graft removal.

## 2. Materials and Methods

A structured narrative review informed by a literature search was conducted using PubMed, Web of Science, and Scopus. Publications published between 2016 and 2025 addressing clinical outcomes, surgical techniques, donor and recipient selection, immunosuppressive therapy, and ethical aspects of uterus transplantation were reviewed. The search strategy combined the terms “uterus transplantation”, “uterine transplantation”, “uterine factor infertility”, “living donor”, “deceased donor”, “pregnancy outcomes”, and “immunosuppression”. The search strategy was adapted as appropriate for each database. Publications addressing clinical outcomes, surgical techniques, donor and recipient selection, immunosuppressive therapy, ethical aspects, and the historical development of uterus transplantation were considered for inclusion.

Duplicate records identified across databases were removed prior to screening. Titles and abstracts were independently screened by two authors (Ž.S.-B. and K.K.). Potentially eligible studies subsequently underwent full-text review according to predefined inclusion and exclusion criteria.

Inclusion criteria comprised peer-reviewed English-language publications addressing uterus transplantation, including clinical studies, case series, systematic reviews, ethical analyses, and methodological reports. Studies reporting surgical techniques, donor and recipient selection, immunosuppressive management, reproductive outcomes, ethical considerations, or the historical development of uterus transplantation were eligible for inclusion. Exclusion criteria included conference abstracts without full text, non-English publications, and studies lacking sufficient relevance to the aims of this review.

Data extraction was independently performed by the two authors. Extracted information included study design, donor and recipient characteristics, surgical approaches, immunosuppressive protocols, reproductive and graft-related outcomes, reported complications, and relevant ethical or methodological considerations. Due to the heterogeneity of the available evidence, a qualitative synthesis was performed.

Given the narrative nature of this review and the substantial heterogeneity of the included studies, a formal quality assessment or risk-of-bias evaluation was not performed.

## 3. Results

### 3.1. The Development and Outcomes of Uterus Transplantation Surgery

The development of uterus transplantation evolved gradually over several decades of experimental and clinical research and has ultimately emerged as a viable treatment option for women with absolute uterine factor infertility (AUFI). Historically, in the second half of the 20th century, initial attention was directed toward surgical attempts to transplant ovaries and fallopian tubes in women whose infertility was caused by dysfunction of these organs [5,8]. Although successful pregnancies were achieved in animal models, these procedures failed to gain clinical relevance because of poor graft outcomes and the subsequent introduction of in vitro fertilization [5,8]. Nevertheless, these early experiences provided the conceptual foundation for the development of uterus transplantation as a potential treatment for AUFI [5,8].

Further development of uterus transplantation was based on extensive preclinical studies using animal models. These studies aimed to evaluate graft revascularization, surgical feasibility, and reproductive function following transplantation. In rodent models, successful pregnancies and live births were achieved following uterus transplantation, without significant implantation disorders or increased miscarriage rates compared to pregnancies without a transplanted uterus. However, the clinical relevance of these models is limited due to differences in gestational duration and anatomy compared to humans [8,9]. Due to greater anatomical similarity, more attention was given to sheep models, where allogeneic uterus transplantation combined with immunosuppression resulted in three pregnancies out of five attempts and one live lamb birth. These findings demonstrated that embryo implantation, pregnancy, and live birth could be achieved following allogeneic uterus transplantation in an animal model with physiology more comparable to humans [8,9].

The first clinical attempts of uterus transplantation in humans were isolated, experimental, and marked by uncertainty. The first documented uterus transplantation from a living donor was performed in 2000 in Saudi Arabia on a woman who had lost her uterus due to obstetric complications. Although the procedure was technically successful and initial graft perfusion appeared adequate, severe graft-related complications developed within a few months, including ischemic changes and graft failure, necessitating hysterectomy three months after transplantation [8,9]. Due to progressive necrosis, the uterus was removed three months after transplantation. Histological findings ruled out immunological rejection, suggesting that the main causes of failure were inadequate uterine blood supply and insufficient fixation within the pelvis [8,9].

A breakthrough in uterus transplantation was achieved in 2012–2013 in Sweden, where the first structured clinical uterus transplantation program was implemented. In this program, nine women, eight of whom had MRKH syndrome, received uterus transplants from living donors. Although the procedures were associated with surgical and postoperative complications, including infections, thrombotic events, and mild rejection episodes, these were generally managed successfully [8,9]. The program culminated in the first successful live birth following uterus transplantation in 2014, providing proof of concept that uterus transplantation could restore reproductive function in women with AUFI [6].

Another important milestone was the development of deceased-donor uterus transplantation. The first deceased-donor transplantation was performed in Turkey in 2011, although the resulting pregnancy ended in miscarriage [8,10]. Subsequent programs in the United States, the Czech Republic, and Brazil further advanced this approach. In 2017, the first successful live birth following deceased-donor uterus transplantation was reported in Brazil, demonstrating the feasibility of this donor model and expanding the potential donor pool for uterus transplantation [10].

### 3.2. Contemporary Clinical Outcomes Across Countries

Major uterus transplantation programs and their key achievements are summarized in Table 1. Across published cohorts, graft survival ranges from 70 to 75%: 67% in the Czech Republic, 78% in Sweden, 100% in Germany and 40% in the United States [5,11]. Clinical pregnancy rates approach 70% among recipients with a functioning graft, and live birth rates range from 43% among all recipients to over 80% among recipients with a functioning graft. Vascular thrombosis remains the leading cause of graft loss, while hypertension, preeclampsia, infections, and preterm birth are among the most frequently reported complications.

Although outcomes vary between programs, the overall evidence demonstrates that uterus transplantation is clinically feasible across different healthcare systems and donor models.

Sweden pioneered clinical uterus transplantation and achieved the first successful live birth in 2014. The Swedish program primarily focused on women with MRKH syndrome, and all recipients in the reported robotic transplantation trial had this diagnosis [12]. The program subsequently introduced robotic donor surgery, which reduced donor morbidity and contributed to the advancement of minimally invasive transplantation techniques [11]. Sweden continues to report favorable outcomes, likely reflecting extensive surgical experience and long-term program development [5,11].

Following the first successful live birth in 2017, the United States established a structured multi-center uterus transplantation program. The U.S. experience represents one of the largest published cohorts and has provided important data regarding graft survival, reproductive outcomes, and pregnancy-related complications after uterus transplantation [12,13].

The Czech uterus transplantation program included ten transplantations, equally divided between living and deceased donors, exclusively in women with MRKH syndrome [14]. The program contributed important advances to the field, including the first successful European live birth following deceased-donor uterus transplantation and the first transplantation using a uterus from a nulliparous donor [14].

Turkey and the Middle East and North Africa (MENA) region have contributed to the development of uterus transplantation through both deceased- and living-donor programs. The first uterus transplantation from a deceased donor was performed in Turkey in 2011, representing an important milestone in the field [15]. Subsequent clinical experience led to successful live births in the region, including pregnancies achieved following both living- and deceased-donor transplantation [15,16]. These experiences demonstrated the feasibility of uterus transplantation beyond Europe and North America and expanded clinical experience with alternative donor strategies [15,16].

### 3.3. Global Statistics on Surgeries and Births

According to a 2025 review of Mats Brännström et al., more than 130 uterus transplantations had been performed worldwide between the first successful birth in 2014 and 2025, resulting in over 70 live births [5]. MRKH syndrome remains the most common indication for uterus transplantation. In a 2025 systematic review including 50 recipients from Sweden, the Czech Republic, and the United States, 94% had MRKH syndrome, while the remaining 6% had undergone hysterectomy for reasons such as endometrial carcinoma. The mean age of recipients was 30 years, with the youngest being 20 years old [7].

In this review, 66% of donors were living, while the remaining grafts were obtained from deceased donors. Graft functionality was defined as the onset and persistence of menstruation for at least 6 months postoperatively. According to this criterion, the success rate was 74%, with better outcomes observed in grafts from living donors compared to deceased donors (75% vs. 57.1%), suggesting that donor type influences transplantation success [7]. More than two-thirds of failures were due to arterial or venous thrombosis, and in rare cases due to persistent uterine infections or chronic graft rejection. In the postoperative period, as many as 71.4% of recipients developed vaginal stenosis, along with kidney impairment, cytopenia, and infections, mostly related to immunosuppressive therapy [7]. More recent reports suggest that robotic-assisted donor surgery may reduce perioperative morbidity through shorter operative times and tissue-sparing techniques. However, uterus transplantation remains a highly complex procedure, and the overall process from organ retrieval and graft preparation to transplantation may still require approximately 15 h [5].

Reproductive outcomes (pregnancies and live births) are evaluated from two perspectives: first, the likelihood of achieving pregnancy with a viable graft, and second, the frequency of maternal and perinatal complications after conception. Among 50 recipients, 70.3% achieved at least one clinical pregnancy. The procedure resulted in two successful births in 21.4% of cases and at least one live birth in 43% of recipients. Most reported deliveries were performed before 37 weeks of gestation. Only a small number of congenital anomalies have been reported, including isolated cases of auricular deformity and patent foramen ovale. Overall, most newborns (78.6%) were reported to be healthy at birth. Children were followed up to 2 years of age, with no observed impairments in physical or emotional development [7].

Slightly different results were reported in a 2024 Swedish systematic review analyzing 40 live births following uterus transplantation in 36 women. Approximately 20% of patients developed arterial hypertension, 7.5% experienced gestational diabetes, and 10% had preterm premature rupture of membranes [17]. Most patients (70%) underwent planned cesarean section between 34 and 36 weeks of gestation. Due to preterm delivery, 20% of newborns had low birth weight, which is 2.5 times higher than in the general population. Respiratory distress syndrome developed in 35% of cases, and 16 infants required treatment in a neonatal intensive care unit [17].

Several conclusions can be drawn from recent reviews published in 2024 and 2025. First, the global number of procedures and live births is increasing, with recent sources reporting more than 130 transplantations and over 70 children born [5]. Second, although graft survival rates are relatively high, a significant proportion of failures are caused by vascular complications, and such pregnancies are associated with a higher rate of maternal and neonatal complications compared to the general population [7,17].

### 3.4. Types of Donations: Living and Deceased Donors

#### 3.4.1. Living Donors

To date, most reported uterus transplantations have been performed using living donors, largely because this approach enables comprehensive pre-transplant evaluation and careful surgical planning compared with deceased donor transplantation [17,18]. By 2024, over 90 uterus transplantations had been performed worldwide, the majority from living donors, with 40 of 49 reported live births achieved using this approach. Surgical success rates reach 78% for living donors compared to 58% for deceased donors [18].

Living donors are typically close relatives, most often mothers, allowing for better immunological compatibility and higher HLA matching, which reduces rejection risk and improves early graft function [18]. However, up to 75% of related donors are excluded due to factors such as nulliparity, recurrent miscarriages, obesity, comorbidities, smoking, or uterine scarring [18]. Older donor age may influence negative transplantation outcomes, although the impact of donor menopause status remains an area of ongoing investigation [18].

A key advantage of living donation is the ability to perform detailed preoperative assessment, including obstetric history, imaging, and microbiological testing, and to plan surgery in advance, reducing cold ischemia time [18]. Despite these benefits, uterus retrieval is a complex procedure lasting 10–13 h due to intricate vascular anatomy [18].

A 2024 review reported 59 living donor hysterectomies, with most donors being genetically related to recipients. About 10% required additional surgical intervention, most commonly due to urinary tract injuries, bleeding, or thrombosis, with ureteral injury being the most frequent complication [18]. Long-term risks may include cardiovascular complications, ovarian insufficiency, sexual dysfunction, and, in some cases, induced menopause following oophorectomy [19].

Given that uterus transplantation is not life-saving, living donation requires strict ethical consideration and fully informed consent. Donors must be aware of surgical risks, potential complications, and the absence of parental rights to any resulting child [19].

#### 3.4.2. Deceased Donors

For uterus transplantation from deceased donors, donors must meet brain death criteria while maintaining stable cardiac function, as donation after cardiac death is generally not suitable due to the need for preserved hemodynamics until organ retrieval [19]. Although this model is ethically favorable because it avoids harm to a living donor, its application is limited by strict selection criteria and low donor availability [19,20].

Data from the French National Organ Donation Program (2014–2019) showed that among 4544 potential donors, only a small proportion met eligibility criteria. Using “very ideal” criteria (age 18–35 years, BMI < 25, no abdominal surgery, no infections or comorbidities), only 124 donors were identified, while broader “ideal” criteria (age 18–45 years, BMI < 30, limited surgical history) yielded 936 donors, still insufficient given increasing demand [20,21]. This limitation may necessitate expanding criteria, potentially affecting graft quality and outcomes. Additionally, donor evaluation is less comprehensive than in living donation and often relies on medical records and family-provided information [19,20].

Family consent is essential, as the uterus is classified as a vascularized composite allograft. Ethical concerns surrounding reproductive organ donation and logistical constraints, including organ transport and exclusion of higher-risk donors, further reduce donor availability [18,19,21]. Organ retrieval timing is unpredictable and often secondary to life-saving organs, leading to prolonged cold ischemia time [19,21]. This was demonstrated in an Italian case, where transport-related delays resulted in a mean cold ischemia time of 18.3 h despite strict donor selection [22].

From a surgical perspective, deceased donation offers advantages such as longer vascular pedicles and vaginal cuff, facilitating reconstruction, and shorter retrieval times (60–120 min) compared to living donors [19,21]. However, these benefits are offset by procedural urgency and increased risk of errors due to fatigue [21].

Clinical outcomes indicate higher complication rates, with graft loss reaching 25% and 12 live births reported among the first 24 cases [21]. In an Italian series, one of three recipients required hysterectomy due to thrombosis, while one achieved a live birth at 34 weeks [22]. Although ethically favorable, the deceased donor model alone is insufficient to meet clinical demand. Current evidence suggests that both living and deceased donor programs are likely to remain important components of uterus transplantation, while improvements in donor selection and organ retrieval logistics may help expand donor availability [19,20,21].

### 3.5. The Course of Surgical Procedures

Uterus transplantation is one of the most complex gynecological procedures, involving vascular surgery, pelvic reconstructive surgery, and reproductive medicine. Unlike transplantation of life-sustaining organs, uterus transplantation is functional and temporary, with the primary goal of enabling pregnancy and childbirth. The procedure consists of five main stages: donor surgery, graft preparation and transplantation into the recipient, immunosuppressive therapy, in vitro fertilization, and graft hysterectomy [18,22,23].

#### 3.5.1. Donor Surgery

##### Deceased Donor Surgery

In the case of a deceased donor, uterus retrieval is usually performed during the explantation of other vital organs, such as the heart, kidneys, and liver. The procedure is carried out via a midline laparotomy. During transplantation, the internal iliac arteries and veins are dissected together with their branches supplying the uterus. The cervix and the upper part of the vagina are mobilized from surrounding tissues. The ureters are transected above the point where they cross the uterine artery. After retrieval of abdominal and thoracic organs, the uterus is flushed in situ and then removed. The total duration of the procedure, including the procurement of other organs, is approximately 5–7 h [22].

From a technical perspective, the deceased donor model allows for the retrieval of longer and larger-diameter vascular segments, which facilitates vascular anastomoses with the recipient’s vessels. During the procedure, the ovarian veins are often preserved along with their connections to the uterine venous plexus, which can be utilized to ensure adequate venous outflow of the graft if needed [24]. A disadvantage of this method is the longer cold ischemia time, defined as the period from cessation of blood flow in the donor to reperfusion in the recipient, during which the graft is preserved in a low-temperature solution. Experimental and histological studies suggest that the uterine myometrium may tolerate at least 6 h of ischemia and potentially up to 24 h; however, the clinically acceptable upper limit remains uncertain [24].

##### Living Donor Surgery

Living donor hysterectomy is considered the most technically complex part of the entire transplantation process. In the initial Swedish case series, the duration of this procedure ranged from 10 to 13 h, while in other centers it ranged from 7 to 11 h [22]. During the operation, it is essential to carefully dissect the uterine artery together with the corresponding segment of the internal iliac artery to ensure adequate arterial blood supply to the graft. In addition, one or two deep uterine veins are isolated along with a portion of the internal iliac vein to create suitable venous anastomoses. Venous dissection is technically challenging, as the deep veins are located close to the ureter and have numerous small side branches, increasing the risk of ureteral injury and bleeding [18].

Due to these anatomical features, the most common complications in donors include ureteral injuries, ureterovaginal fistulas, pyelonephritis, and dehiscence of the vaginal cuff sutures [23]. In several established uterus transplantation programs, open donor hysterectomy has increasingly been replaced by robotic-assisted techniques [18,24]. This approach allows for more precise vascular dissection due to three-dimensional visualization, improved surgical field exposure, and greater instrument accuracy, which is particularly important when preparing the deep uterine veins [18]. Published reports suggest that robotic surgery may be associated with reduced intraoperative blood loss, shorter hospital stays, and faster return to normal daily activities for donors [23]. However, robotic surgery is not without limitations. A prolonged learning curve and increased operative time in the early stages may contribute to complications [18,24].

#### 3.5.2. Recipient’s Surgery

The recipient surgery is generally shorter than donor hysterectomy; however, it remains technically complex due to the need for precise vascular anastomoses in small-diameter vessels and adequate reconstruction of pelvic anatomy. Depending on the case series, the duration of the recipient procedure ranges from 2 to 6 h, with an average of 4–5 h [18,22]. The operative time depends on the origin of the graft (living or deceased donor), the length and diameter of the vessels, the recipient’s anatomy, and any previous surgical history. The procedure begins with the preparation of the external iliac vessels and separation of the vaginal cuff from the bladder and surrounding tissues. In cases of MRKH syndrome, rudimentary uterine tissue is removed up to the level of the vaginal cuff prior to implantation [18]. The graft is then positioned in the pelvis, restoring the natural anatomical position of the uterus. Arterial inflow is established by connecting the donor’s vessels to the lateral wall of the recipient’s vessels. Venous outflow is created by anastomosing segments of the donor’s internal iliac vein or deep uterine veins to the recipient’s external iliac vein [22]. In some cases, to optimize venous drainage, the ovarian veins may also be utilized if they were preserved in the donor [18].

After restoration of blood flow, vaginal anastomosis is performed by connecting the graft vaginal edge to the recipient’s vaginal cuff, and the uterus is further stabilized using ligaments [18]. Most reported early graft losses are associated with vascular thrombosis or impaired uterine perfusion, which may be related to factors such as small vessel diameter, technical challenges during vascular anastomosis, or pre-existing vascular changes in the donor [18]. In the later postoperative period, complications such as vaginal stenosis may occur due to size mismatch between the donor and recipient vaginal structures [18]. Postoperative evaluation is performed using Doppler ultrasound to assess uterine arterial blood flow, monitor uterine size, and evaluate endometrial proliferation, with the expectation of the onset of menstruation. The return of regular menstruation is generally considered an important clinical indicator of graft viability and function, although comprehensive assessment also includes imaging findings and clinical evaluation [18].

#### 3.5.3. In Vitro Fertilization and Pregnancy Strategy

Natural conception after transplantation is not possible, as the uterus is transplanted without fallopian tubes. Therefore, in vitro fertilization (IVF) becomes an essential part of the process. Preparation for fertilization begins prior to transplantation in order to reduce the duration of systemic immunosuppression in the recipient. Oocyte retrieval and embryo creation are performed before surgery, and embryos are cryopreserved until the graft becomes functionally ready for implantation [22,23]. IVF prior to transplantation is performed to avoid complications such as ovarian hyperstimulation syndrome or infections following oocyte retrieval, which could complicate the early postoperative period. The exact probability of graft survival remains uncertain. Before undertaking a high-risk procedure, it is important to ensure that the recipient has an adequate embryo reserve to achieve a realistic chance of pregnancy. Although no universal consensus exists regarding the optimal number of cryopreserved gametes or embryos, several centers aim to obtain at least 20 unfertilized oocytes or 6–8 blastocysts before transplantation to maximize the chance of achieving a live birth [22]. Despite these recommendations, cases of embryo depletion are observed in clinical practice. In U.S. centers, approximately 20% of recipients face a shortage of embryos and require repeat oocyte retrieval after transplantation [23].

In early uterus transplantation programs, embryo transfer was commonly delayed for approximately one year after surgery to optimize immunosuppressive therapy and confirm graft functionality. More recent reports suggest a trend toward shorter intervals, often 3–6 months after transplantation, provided that graft perfusion is normal and no rejection episodes have occurred; however, protocols continue to vary between centers [23].

Pregnancy following uterus transplantation is generally considered high-risk and is typically managed in tertiary-level healthcare centers with expertise in maternal-fetal medicine and transplantation [22,23]. Risk factors include not only the effects of immunosuppression but also an increased risk of preeclampsia and preterm delivery [22,23]. Most uterus transplantation programs perform planned cesarean delivery to minimize potential risks to the graft and because cervical function may be compromised following transplantation and associated surgical procedures [22,23]. In some recipients, cervical function is impaired due to prior surgical interventions, which precludes vaginal delivery. The timing of delivery varies between centers; however, many programs favor planned cesarean delivery between 35 and 37 weeks of gestation when clinical conditions remain stable [22].

#### 3.5.4. Hysterectomy of the Transplant

Unlike other organ transplantations, the ultimate goal in uterus transplantation is not long-term organ survival, but temporary functional use to achieve reproductive outcomes. Planned graft removal after completion of reproductive goals is a rational strategy because it reduces long-term exposure to immunosuppressive therapy and minimizes associated health risks. Consequently, uterus transplantation is generally regarded as a temporary, function-oriented procedure [18,22]. In many reported uterus transplantation programs, graft hysterectomy is performed after completion of reproductive goals, commonly following one to three successful pregnancies or approximately 5–7 years after transplantation to minimize long-term exposure to immunosuppressive therapy; however, the timing of graft removal remains center-dependent and is individualized according to reproductive plans and clinical circumstances [18,22]. From a surgical perspective, graft hysterectomy is not equivalent to a standard gynecological hysterectomy. During the procedure, previously created arterial and venous anastomoses between the graft and the recipient’s iliac vessels must be identified, dissected, and ligated. Due to postoperative adhesions and altered pelvic anatomy, this part of the procedure may be technically more challenging than the initial implantation [22].

Depending on the clinical situation and the center’s experience, graft removal may be performed either during the cesarean section in the final pregnancy or as a separate procedure after delivery [22]. In addition to planned removal, less common indications for hysterectomy include severe bacterial, viral, or fungal infections, as well as uterine abscesses and septic complications. In such cases, graft removal becomes a life-saving intervention to protect the recipient’s health [18]. Published evidence suggests that graft loss due to treatment-resistant acute rejection is rare, whereas most early graft removals are related to surgical or vascular complications rather than immunological incompatibility [23].

### 3.6. The Significance of Immunosuppression and Associated Risks

Immunosuppressive therapy is essential to ensure graft viability and achieve the primary goal of the procedure—successful pregnancy and live birth. In the absence of dedicated uterus transplantation guidelines, immunosuppressive protocols are commonly adapted from kidney transplantation practice. Induction therapy is initiated with anti-thymocyte globulin or the interleukin-2 receptor antagonist basiliximab in combination with corticosteroids, while maintenance therapy is based on tacrolimus, most combined with azathioprine or mycophenolate mofetil [25,26]. The use of mycophenolate mofetil is limited due to its teratogenic effects; therefore, it is discontinued or replaced with azathioprine prior to embryo transfer. The immunosuppressive regimen is adjusted to ensure both maternal and fetal safety [3,22]. There are no universally accepted guidelines for immunosuppressive therapy, and therefore practices vary between centers. In a multicenter study from the United States and Europe, anti-thymocyte globulin was more commonly used for induction, whereas the Singapore program preferred basiliximab due to a lower risk of immunological complications, along with strict monitoring of tacrolimus levels [26]. The intensity of immunosuppression may be individualized based on the recipient’s immunological profile and the center’s experience. Uterus grafts are characterized by frequent rejection episodes; therefore, protocol-based monitoring with cervical biopsies is required. Clinical symptoms of rejection are often minimal or absent. Most rejection episodes are cellular in nature and respond well to pulse corticosteroid therapy. Although rejection episodes are relatively common, graft loss due solely to immunological rejection appears to be rare [3,23].

A multicenter analysis including uterus transplantations performed between 2016 and 2020 in the United States and Europe, involving 18 women who achieved at least one live birth, identified nephrotoxicity as the most significant complication of systemic immunosuppression. All recipients demonstrated a reduction in glomerular filtration rate associated with tacrolimus nephrotoxicity: the mean eGFR decreased from 107 mL/min/1.73 m^2^ before transplantation to 87 mL/min/1.73 m^2^ 30 days after surgery (approximately a 20 mL/min decrease) and remained lower than baseline at the time of embryo transfer (94 mL/min/1.73 m^2^) [25]. During pregnancy, approximately half of the recipients developed stage I acute kidney injury according to AKIN criteria [25]. Although renal function improved in some women after graft removal and discontinuation of immunosuppressive therapy, long-term renal outcomes remain unclear [3,25].

Due to the suppression of cellular and humoral immunity associated with immunosuppressive therapy, infectious complications represent a significant risk after uterus transplantation. Consequently, antimicrobial, antifungal, and antiviral prophylaxis are commonly incorporated into postoperative management protocols, although specific regimens vary between centers. Frequently used agents include broad-spectrum antibiotics, antifungal therapy (e.g., anidulafungin or nystatin), and antiviral prophylaxis with ganciclovir or valganciclovir. Particular attention is paid to the prevention and monitoring of cytomegalovirus (CMV) infection, which remains one of the most important infectious concerns in transplant recipients [26].

During pregnancy, immunosuppressive therapy remains necessary to maintain graft function and is most based on tacrolimus, azathioprine, and low-dose corticosteroids. Drug levels and clinical parameters require regular monitoring to ensure both maternal and fetal safety [3,22]. Clinical follow-up includes surveillance of blood pressure, renal function, and infectious complications, together with continuous assessment of fetal well-being [3].

### 3.7. Adverse Events and Complications Associated with Uterus Transplantation

Uterus transplantation is associated with complications affecting donors, recipients, graft function, and pregnancy. The most common causes of graft failure are vascular complications, particularly arterial and venous thrombosis, while less frequent causes include persistent uterine infections and chronic graft rejection. In the postoperative period, some recipients may develop vaginal stenosis, and immunosuppressive therapy may lead to nephrotoxicity, cytopenias, and infectious complications. Living donor surgery is associated with considerable operative morbidity due to its technical complexity. Approximately 10% of donors require additional surgical intervention, most commonly because of ureteral injury, urinary tract complications, bleeding, or thrombosis. Long-term donor risks may include cardiovascular complications, ovarian insufficiency, sexual dysfunction, and, in selected cases, menopause following oophorectomy. Pregnancy after uterus transplantation remains high-risk and is associated with increased rates of hypertensive disorders (approximately 20%), gestational diabetes (7.5%), preterm premature rupture of membranes (10%), and preterm delivery. Consequently, approximately 20% of newborns have low birth weight, respiratory distress syndrome occurs in around 35%, and a substantial proportion require neonatal intensive care. Although most reported complications are manageable in experienced multidisciplinary centers, they continue to represent one of the principal limitations of uterus transplantation and emphasize the importance of careful patient selection, meticulous surgical technique, and comprehensive long-term follow-up [7,17,19,22,23,25,26].

### 3.8. Ethical Aspects: Donor Safety and Consent

The ethical aspects of uterus transplantation related to donor safety and informed consent are more complex than in traditional solid organ transplantation due to its non-lifesaving, quality-of-life–enhancing nature [7,27]. Because of the strict medical and psychosocial selection criteria applied to living donors, some centers have considered altruistic (non-directed) donation as a potential strategy to expand the donor pool. However, this approach raises additional ethical concerns regarding donor motivation, voluntariness, and the potential for coercion or commercialization [27,28]. Justifying surgical risk requires strict criteria, as harm to a healthy individual cannot be equated with life-saving organ donation. In living donation, the donor undergoes a long and technically demanding procedure, with possible complications including infections, ureteral injury, fistulas, urinary dysfunction, and chronic pain. Early reports indicate higher rates of ureteral injury compared to standard hysterectomy [27].

Long-term risks may also affect quality of life. Although ovaries are typically preserved, impaired ovarian blood supply may lead to premature ovarian insufficiency and early menopause requiring hormone replacement therapy [28]. Psychological risks are equally significant. Related donors may experience pressure or a sense of obligation, and emotional distress may arise in cases of transplant failure or changing personal circumstances. Although most donors report favorable psychological outcomes, evidence from living organ donation indicates that a minority may experience significant psychological difficulties. In a U.S. cohort study, approximately 4% of living kidney donors reported substantial psychological distress following donation [28].

The use of deceased donors eliminates the ethical issue of exposing a healthy individual to harm but introduces challenges related to consent and autonomy [27]. General organ donation consent should not be assumed to include uterus donation, as the uterus holds symbolic reproductive significance. Like face or limb transplantation, specific consent may be required. Additionally, reliance on family authorization may not accurately reflect the donor’s wishes due to limited public awareness [28].

Since uterus transplantation improves quality of life rather than saving lives, it must not interfere with the allocation of life-saving organs. Uterus procurement should only proceed when it does not compromise other transplant outcomes [28].

In living donation, informed consent must be supported by thorough medical and psychological evaluation. Donors must be fully informed and free to withdraw at any stage. Decision-making is conducted by a multidisciplinary team assessing both medical risk and psychological readiness. Procedures are declined if risks are excessive or autonomy is uncertain [22,27,28].

Current ethical discussions on uterus transplantation are largely centered around two perspectives. The first emphasizes the principle of non-maleficence and questions whether exposing a healthy individual to the risks of major surgery can be ethically justified for a procedure that improves quality of life rather than saves life [27]. The second perspective supports living donation when donor risks are minimized, informed consent is fully voluntary and comprehensive, and rigorous medical and psychological evaluation is performed. Proponents of this view argue that uterus transplantation can be ethically acceptable when conducted within specialized programs that prioritize donor safety and continuously seek to reduce reliance on living donors through the expansion of deceased-donor programs and the development of alternative approaches [27,28].

## 4. Discussion

Uterus transplantation remains an innovative and highly specialized treatment method for AUFI [6,7]. Over the past decade, more than 130 uterus transplantations have been performed worldwide, resulting in over 70 reported live births [5,7].

Unlike conventional solid-organ transplantation, uterus transplantation is not a life-saving procedure but rather a quality-of-life intervention aimed at restoring reproductive potential. This unique characteristic substantially influences recipient selection, donor evaluation, ethical considerations, and the acceptable balance between procedural risks and expected benefits. Consequently, clinical decision-making in uterus transplantation requires a multidisciplinary approach that extends beyond traditional transplant medicine [6,7].

Surgical factors play a central role in determining transplantation success. Graft survival reaches approximately 74%, with more than two-thirds of failures attributed to vascular complications, particularly thrombosis [7,29]. Better outcomes are observed with living donors (75% vs. 57.1%), although this raises ethical concerns regarding donor safety [7,19].

One of the major unresolved questions concerns the optimal donor source. Living donors provide the advantages of comprehensive preoperative assessment, planned surgery, and shorter ischemia times, which may contribute to improved graft survival. However, this benefit must be weighed against the ethical concerns associated with exposing healthy individuals to a major surgical procedure. In contrast, deceased donation eliminates donor morbidity but remains limited by donor availability, logistical challenges, and prolonged ischemia times. Current evidence suggests that both approaches remain clinically relevant, and future programs will likely continue to rely on a combination of donor models [18,19,20,21].

Despite increasingly encouraging reproductive outcomes, uterus transplantation remains one of the most resource-intensive procedures in reproductive medicine. The requirement for complex surgery, prolonged follow-up, immunosuppressive therapy, and multidisciplinary expertise raises important questions regarding cost-effectiveness and accessibility. At present, the procedure is available only in highly specialized centers, limiting access for most patients with absolute uterine factor infertility. Broader implementation will depend not only on improving clinical outcomes but also on reducing procedural complexity and resource requirements [6,23].

Reproductive outcomes are promising; however, pregnancies remain high-risk. Clinical pregnancy is achieved in approximately 70.3% of recipients, while at least one live birth occurs in 43% [7]. Common complications include hypertension (~20%), gestational diabetes (7.5%), and preterm delivery (34–36 weeks). Newborns more frequently present with low birth weight (20%) and respiratory distress (35%) [18].

Immunosuppression remains essential but is associated with significant complications, particularly nephrotoxicity and infections, and the optimal safe regimen has not yet been established [6]. Given that recipients are typically young and otherwise healthy women, long-term risk assessment remains crucial. Available follow-up data on children born after uterus transplantation are generally reassuring, with most studies reporting normal physical growth and neurodevelopment during early childhood. However, the number of offspring remains limited, and follow-up periods are relatively short. Long-term monitoring into adolescence and adulthood will be necessary to fully evaluate the safety of uterus transplantation for future generations.

Interpretation of the available evidence is limited by several methodological challenges. Most published uterus transplantation studies originate from a small number of highly specialized centers and include relatively small patient cohorts. In addition, overlap between primary studies and subsequent follow-up reports may complicate interpretation of cumulative outcomes. Comparisons between programs are further hindered by differences in donor selection, surgical techniques, immunosuppressive protocols, and outcome definitions, particularly regarding graft survival, clinical pregnancy, and live-birth rates. Consequently, direct comparison of reported success rates should be interpreted with caution.

The main limitations in this field include small sample sizes, heterogeneous methodologies, and a lack of long-term data [6]. Future developments are expected to focus on three principal areas: refinement of surgical techniques to reduce donor morbidity, optimization of immunosuppressive protocols to minimize maternal and fetal risks, and advancement of tissue-engineered or bioengineered uterus constructs. The latter approach is particularly promising because it could potentially eliminate the need for living or deceased donors while reducing the ethical and immunological challenges currently associated with uterus transplantation [6,23].

## 5. Conclusions

Current evidence indicates that uterus transplantation can restore reproductive function in carefully selected women with absolute uterine factor infertility, with graft survival rates of approximately 74%, clinical pregnancy rates approaching 70%, and more than 70 live births reported worldwide. Nevertheless, the procedure remains highly specialized and is associated with significant surgical, vascular, and immunological risks. Future progress will depend on improving graft survival, reducing donor morbidity, and generating robust long-term data on recipient, graft, and offspring outcomes.

## Figures and Tables

**Table 1:** Major uterus transplantation programs and key achievements.

Program	Recipient Population	Donor Type	Transplants (*n*)	Key Achievements
Sweden	MRKH syndrome	Living donor	8	First successful live births; robotic donor surgery
USA	Predominantly MRKH syndrome	Predominantly living donor	33	Largest reported cohort; 21 live births
Czech Republic	MRKH syndrome	Living and deceased donor	10	First European live birth after deceased-donor UTx
Turkey/MENA	Predominantly MRKH syndrome	Living and deceased donor	Case series	First deceased-donor UTx; successful births
Global experience	Mostly MRKH syndrome	Mixed	>130	>70 live births reported

## Data Availability

No new data were created or analyzed in this study.
